# Protector Role of Cx30.2 in Pancreatic β-Cell against Glucotoxicity-Induced Apoptosis

**DOI:** 10.3390/biology13070468

**Published:** 2024-06-25

**Authors:** Daniel Ortega-Cuellar, Ignacio González-Sánchez, Gabriela Piñón-Zárate, Marco A. Cerbón, Víctor De la Rosa, Yuliana Franco-Juárez, Andrés Castell-Rodríguez, León D. Islas, Cristina Coronel-Cruz

**Affiliations:** 1 Laboratorio de Nutrición Experimental, Instituto Nacional de Pediatría, Secretaría de Salud, Mexico City 04530, Mexico; dortegadan@gmail.com (D.O.-C.); yulianafjbio@gmail.com (Y.F.-J.); 2 Departamento de Biología, Facultad de Química, UNAM, Mexico City 04510, Mexico; igonzalez@quimica.unam.mx (I.G.-S.); mcerbon85@yahoo.com.mx (M.A.C.); 3 Departamento de Biología Celular y Tisular, Facultad de Medicina, UNAM, Mexico City 04510, Mexico; gabrielapinon@unam.mx (G.P.-Z.); castell@unam.mx (A.C.-R.); 4 Departamento de Fisiología, Facultad de Medicina, UNAM, Mexico City 04510, Mexico; victor.de.la.rosa.j@gmail.com (V.D.l.R.); leon.islas@gmail.com (L.D.I.)

**Keywords:** insulin-secreting cells, glucotoxicity, connexin 30.2, apoptosis, gap junction channels

## Abstract

**Simple Summary:**

Glucotoxicity is a condition that leads to β-cell death, driving a decrease in insulin secretion and leading to hyperglycemia and, eventually, diabetes. Here, we investigate whether connexin Cx30.2 could protect against glucotoxicity-induced apoptosis in β cells. We found that RIN-m5F β cells exposed to high glucose exhibited increased Cx30.2 protein expression. Interestingly, decreased expression of Cx30.2 by specific siRNAs resulted in augmented β-cell death, suggesting that Cx30.2 has an important role in maintaining cell survival during glucotoxicity. Overall, Cx30.2 could be an attractive pharmacological target to avoid high glucose-induced β-cell apoptosis.

**Abstract:**

Glucotoxicity may exert its deleterious effects on pancreatic β-cell function via a myriad of mechanisms, leading to impaired insulin secretion and, eventually, type 2 diabetes. β-cell communication requires gap junction channels to be present among these cells. Gap junctions are constituted by transmembrane proteins of the connexins (Cxs) family. Two Cx genes have been identified in β cells, Cx36 and Cx30.2. We have found evidence that the glucose concentration on its own is sufficient to regulate Cx30.2 gene expression in mouse islets. In this work, we examine the involvement of the Cx30.2 protein in the survival of β cells (RIN-m5F). Methods: RIN-m5F cells were cultured in 5 mM D-glucose (normal) or 30 mM D-glucose (high glucose) for 24 h. Cx30.2 siRNAs was used to downregulate Cx30.2 expression. Apoptosis was measured by means of TUNEL, an annexin V staining method, and the cleaved form of the caspase-3 protein was determined using Western blot. Results: High glucose did not induce apoptosis in RIN-m5F β cells after 24 h; interestingly, high glucose increased the Cx30.2 total protein levels. Moreover, this work found that the downregulation of Cx30.2 expression in high glucose promoted apoptosis in RIN-m5F cells. Conclusion: The data suggest that the upregulation of Cx30.2 protects β cells from hyperglycemia-induced apoptosis. Furthermore, Cx30.2 may be a promising avenue of therapeutic investigation for the treatment of glucose metabolic disorders.

## 1. Introduction

Pancreatic β cells act as glucose sensors, adjusting insulin secretion to the prevailing blood glucose level, thus maintaining glucose homeostasis [1]. The deterioration of β-cell function and the concomitant damaged insulin secretion results in chronic hyperglycemia, which characterizes type 2 diabetes, currently affecting about 422 million people worldwide [2]. Glucose induces negative effects on β-cell function when present in excessive concentrations over a chronic period, causing apoptosis [3,4,5,6,7].

Connexins (Cxs) are a transmembrane protein family; about twenty connexin isoforms are expressed in mammal cells [8]. Structurally, six Cxs form a connexon or hemichannel, and two connexons, each provided by an adjacent cell, constitute intercellular channels named gap junction (GJ) channels, which allow for intercellular communication among adjacent cells and can pass ions, nutrients, second messengers, and other metabolites of less than 1 kDa [9,10,11]. GJ facilitates the coordination of electrical activity across the cells [12]. Since channels and hemichannels formed by Cxs also allow for the exchange of ions and molecules between the inside and outside of the cell, they are also seen as mediators of tissue homeostasis [13,14,15,16]. It is widely documented that GJ channels, through the passage of multiple signaling molecules, can modulate cell growth and apoptotic cell death, depending on the cellular context [17,18]. 

It has been demonstrated that the isoform Cx36 is mainly expressed in β cells [19,20,21]. Cx36 can provide electrical coupling between β cells to promote insulin secretion [22,23,24,25,26]. For a long time, it was thought that Cx36 was the only connexin expressed in β cells. However, more than a decade ago, we demonstrated that Cx30.2 is also expressed in pancreatic β cells in mice, where protein co-localizes with Cx36 at junctional membranes in β cells. The role of Cx30.2 in the intercellular communication of β cells is still unknown. Additionally, Cx30.2 mRNA expression was reduced when cultured in mouse islets with high glucose (22 mM) [27], suggesting that Cx30.2 protects β cells against glucotoxicity. Consistent with this, Cx30.2 was also downregulated in the retinal capillaries of diabetic rats, which increased their risk of diabetic retinopathy, demonstrating that Cx30.2 has a protective role in combating glucotoxicity [28]. Similarly, the reduction of Cx36 increased apoptosis in β cells exposed to proinflammatory cytokines similar to those found in type 1 diabetes. In contrast, overexpressed Cx36 leads to β-cell survival [29]. Interestingly, the overexpression of Cx36 was able to protect islets from ER and oxidative stress, and apoptosis induced by pro-inflammatory cytokines [30]. Moreover, in β-cell INS-1E and pancreatic islets exposed to oxidized low-density lipoproteins, an environment similar to that in type 2 diabetes, Cx36 decreased, inducing apoptosis [31]. These studies suggest a function of Cxs in coping with adverse conditions for β cells. 

Since we discovered that high glucose decreased the Cx30.2 transcript in islets, this led us to hypothesize that Cx30.2 might play an important role in the glucotoxic deterioration of the β-cell phenotype. This study aimed to determine the function of Cx30.2 in response to high glucose levels of insulinoma β cells to establish its anti-apoptotic effects.

## 2. Materials and Methods

### 2.1. Cell Line and Culture Conditions

RIN-m5F cells were purchased from ATCC^®^ (CRL 11605™, Manassas, VA, USA). The cells were grown in RPMI 1640 with 11 mM glucose (Sigma, St. Louis, MO, USA) supplemented with 10% fetal bovine serum (Gibco, Grand Island, NY, USA) and penicillin/streptomycin (Gibco, NY, USA) in a humidified atmosphere consisting of 5% CO_2_ at 37 °C. 

### 2.2. Immunofluorescence Assays

A total of 1 × 10^5^ RIN-m5F cells were cultured in 24-well plates with round cover glasses in RPMI with 5 mM glucose or 30 mM glucose for 24 h. Then, the cells were washed with PBS and fixed with 4% paraformaldehyde for 10 min. The cells were placed in a blocking buffer for 1 h and incubated overnight with polyclonal antibody rabbit anti-Cx30.2 (Cat. 40-7400, Invitrogen, Carlsbad, CA, USA). After rinsing, they were incubated for 1.5 h at room temperature with a secondary antibody donkey anti-rabbit (Cat. Ab96921, Abcam, Cambridge, UK). Immunofluorescence images were taken using a confocal laser scanning microscope (Olympus model FV1000, Tokyo, Japan).

### 2.3. Western Blot

A total of 2 × 10^6^ RIN-m5F cells were cultured in RPMI 1640 medium with 5 mM glucose or 30 mM glucose for 24 h. The proteins were extracted using buffer RIPA (cat. 20-188, Millipore, Volketswil, Switzerland) supplemented with protease inhibitors (Cat. P8340, Sigma) and homogenized using sonication. The supernatant was separated using centrifugation (15,000 rpm for 15 min at 4 °C) and the proteins were quantified using the BCA method. Then, 50 μg of the total protein was loaded and separated using SDS-PAGE on a 12% polyacrylamide gel and transferred to a PVDF membrane (Bio-Rad Laboratories, Hercules, CA, USA). The membrane was placed in blocking buffer (5% fat-free milk in TBS-Tween 20 buffer) for 1 h and incubated overnight with polyclonal antibodies: rabbit anti-Cx30.2 (Cat. 40-7400, Invitrogen) or the 17 KDa cleaved form, anti-caspase 3 (Cat. ab13847, Abcam), or rabbit anti-actin (Cat. A2103, Sigma, St. Louis, MO, USA), which was used as the loading control. After rinsing, they were incubated for 1 h at room temperature with a horseradish peroxidase (HRP) secondary antibody. The membranes were revealed using chemiluminescence assays, and the protein of interest was analyzed using Quantity-One software (Version 4.6.8) (Bio-Rad Laboratories, Hercules, CA, USA).

### 2.4. TUNEL Assay

A total of 1 × 10^5^ RIN-m5F cells were cultured in 24-well plates with round cover glasses in RPMI with 5 mM glucose or 30 mM glucose for 24 h. Some cells were incubated with 125 nM taxol for 72 h for a positive control of apoptosis. The cells were washed with PBS and then fixed with 4% paraformaldehyde for 10 min. DNA fragmentation was evaluated using the TUNEL assay (In Situ Cell Death Detection kit, Cat. 12156792910, Roche, Mannheim, Germany), according to the manufacturer’s instructions. After fixation, the cells were permeabilized with 0.1% Triton X-100 in 0.1% sodium citrate and then rinsed twice with PBS. The DNA nick-labeling reaction was performed at 37 °C for 60 min using 50 μL of the TUNEL reaction mixture, which included 45 μL of labeled nucleotide mix and 5 μL of enzyme solution. After incubation, the samples were rinsed with PBS, and the nuclei were dyed with DAPI and washed three times with PBS. Then, they were placed on slides using Vectashield Mounting Medium (Vector, San Mateo, CA, USA). The fluorescence was analyzed and recorded using a confocal laser scanning microscope (LSM 880, Zeiss, Oberkochen, Germany). Quantification of the TUNEL-positive cells was realized using the ImageJ software V1.54 (NIH, Bethesda, MD, USA).

### 2.5. Flow Cytometry Assay of Apoptosis

A total of 5 × 10^5^ Rin-m5F cells were cultured in 12-well plates with 5 mM glucose or 30 mM glucose for 24 h or with 125 nM taxol for 72 h. The latter was used as a positive control of apoptosis. Then, the cells were washed with PBS, and detached using trypsin (0.05%). The cells were collected using centrifugation, rinsed with 500 µL of BioLegend’s cell staining buffer, and suspended in a 100 µL binding buffer. The Annexin V/7-AAD Apoptosis Detection Kit (Cat. 640912, BioLegend, San Diego, CA, USA) was used for flow cytometry analysis of the apoptotic cells. Each sample was stained with 5 µL annexin V-FITC and 5 µL 7-AAD for 15 min, and finally, 400 µL binding buffer was added. Data acquisition was performed in a FACScalibur cytometer at the National Laboratory of Flow Cytometry (LabNalCit, Biomedical Research Institute, Noda, Japan), and analysis was performed using FlowJo software, 10.4.0.

### 2.6. Cell Viability

A total of 1.4 × 10^4^ RIN-m5F cells were seeded on 96-well plates in RPMI with 5 mM glucose or 30 mM glucose for 24 h, or 125 nM taxol for 72 h, which was used as a negative control of viability. The effect of glucose concentration on cell metabolism was determined using an MTT assay, performed as previously reported [32]. After 24 h of incubation, the cells were exposed to 20 µL of MTT (2.5 mg/mL) and incubated for 1.5 h at 37 °C. Then, the supernatant was removed, and the formed insoluble formazan crystals were dissolved in 100 µL of DMSO. The absorbance of each well was measured at 540 nm using an Epoch Microplate Spectrophotometer (Biotek^®^, Winooski, VT, USA). The cell viability or cell metabolism was determined by comparing the absorbance of the cells cultured with 30 mM glucose or taxol with respect to the absorbance of the cells in 5 mM glucose, corresponding to 100% viability.

### 2.7. siRNAs Transfection

For the siRNA experiments, RIN-m5F cells were transfected with Cx30.2 specific-siRNAs mix (Cat. D-085943-01-0005 and D-085943-02-0005), or nonspecific siRNA siGENOME non-targeting (scramble siRNA, from Dharmacon, Lafayette, CO, USA) was used as a control. The cells were transfected with DharmaFECT transfection reagent (Dharmacon) and 75 nM of Cx30.2-siRNAs mix or the non-targeting control siRNA per plate according to the manufacturer’s protocol. After 24 h, the transfection medium was removed, and TUNEL and flow cytometry assays were performed.

### 2.8. Scrape Loading/Dye Transfer Assay (SLDT)

To measure the activity of the GJ intercellular channels (GJIC), RIN-m5F cells were grown in 35 mm dishes up to 90% confluence with 5 mM glucose or 30 mM glucose for 24 h. To determine the effects of high glucose on the GJIC activity, an SLDT assay was performed, following the procedure described previously [33]. Briefly, following treatment, the RPMI was removed, and the cells were washed with PBS containing 0.01% Ca^2+^ and Mg^2+^. Next, three random cuts were made to the monolayer of cells using a scalpel blade in the presence of DAPI solution (0.05 mg/mL), and they were incubated for 5 min to allow for the transfer of the DAPI to the neighboring cells (at room temperature under minimum illumination). Then, the cells were washed three times with PBS, fixed with 4% paraformaldehyde, and photographed with a digital camera (Nikon Digital Sight; Miyagi, Japan) attached to a fluorescence microscope (Nikon Eclipse E600, Miyagi, Japan). The GJ activity was observed from the transference of the DAPI from the site cut to the adjacent cells in ten different regions under each glucose condition. Quantification of the number of dye-coupled cell layers was carried out using ImageJ software V1.54 (NIH, Bethesda, MD, USA).

### 2.9. Dye Transfer Studies

A total of 1 × 10^6^ RIN-m5F cells were cultured in a 35 mm cell culture dish in RPMI medium with 5 mM glucose or 30 mM glucose for 24 h. The cells were patch-clamped and dialyzed with DAPI (0.05 mg/mL) dissolved in a physiological internal solution containing: 140 mM KCl, 10 mM EGTA, 2 mM MgCl_2_, 10 mM HEPES, and 10 mM glucose, pH 7.3. The external Ringer’s solution contained: 145 mM NaCl, 5 mM KCl, 2 mM CaCl_2_, 1 mM MgCl_2_, 10 mM HEPES, and 10 mM glucose, pH 7.4. Membrane resistance was monitored with an Axopatch 200B patch clamp amplifier (Axon Instruments, San José, CA, USA) and digitized with an ITC-18 computer interface (HEKA Electronik) controlled using Patchmaster software V2x91 (HEKA Electronik, Holliston, MA, USA). Fluorescence measurements were made using a TE-200U inverted microscope with a 40X objective. A 405 nm solid-state laser (Compass 405-50 CW, Coherent, Saxonburg, PA, USA) and a filter cube containing a 405/20 nm excitation filter, a 405 nm long pass dichroic mirror, and a 425 nm long-pass emission filter were used for DAPI fluorescence excitation. Imaging was performed using an Ixon Ultra EMCCD camera (Oxford Instruments, Abingdon, UK, Ireland) controlled with Micromanager software 1.4.21. The cells were held at −70 mV during the whole experiment and left for 10 to 20 min for the dye transfer. Images were taken every 2 min and analyzed offline using ImageJ software V1.54 (NIH, Bethesda, MD, USA). Membrane resistance was monitored as an indicator of membrane integrity.

### 2.10. Statistical Analysis

The data are reported as the mean ± standard error of the mean of three independent experiments, with six replicates each. Statistical analysis was performed using GraphPad Prism 8 (GraphPad Software, La Jolla, CA, USA). Statistically significant differences between the two treatments were analyzed with Student’s *t*-test. *p* ≤ 0.05 was considered to indicate statistical significance.

## 3. Results

### 3.1. High Glucose Exposure Did Not Promote RIN-m5F Cells Apoptosis

**Figure 1:**
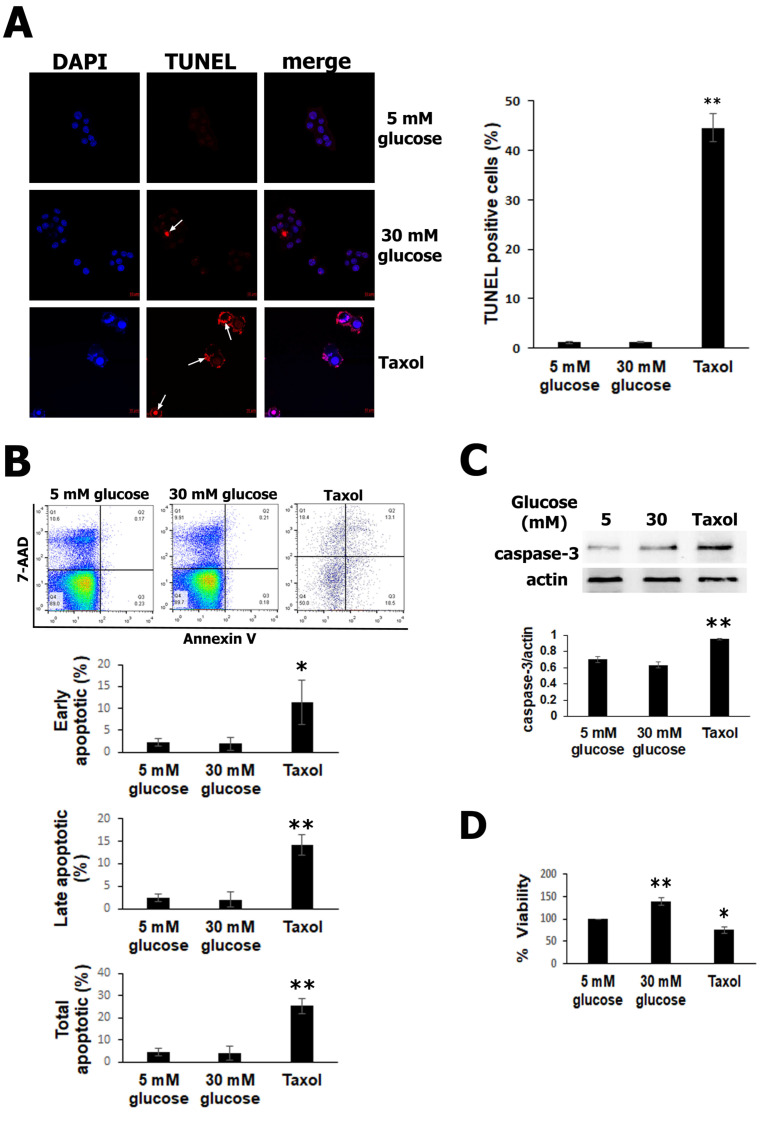
Elevated glucose does not induce apoptosis in RIN-m5F cells. (**A**) TUNEL assays detected significantly increased apoptotic cells exposed to taxol, used as a positive control (intense red mark, white arrows). (**B**) Population diagrams showing flow cytometry using an annexin V kit, which did not detect early or late apoptosis with high glucose compared to normal glucose. In contrast, the taxol group was used as a positive control of apoptosis, showing a significant increase. (**C**) The detection of cleavage of caspase-3 using Western blot demonstrated that there were no differences in active caspase-3 between the 5 mM and 30 mM glucose groups; however, in the taxol group, cleaved caspase-3 was augmented. (**D**) MTT assays showed that cell metabolism increased with 30 mM glucose compared to normal glucose and was reduced in the taxol group, which was later used as a negative control. The data show the mean values ± SE. * *p* ˂ 0.05 and ** *p* ˂ 0.01 represent significant differences among the treatments from three independent experiments.

It has been well documented that high glucose can trigger cell death via apoptosis [7,34,35]. To determine whether high glucose can negatively affect cell survival, RIN-m5F cells were exposed to 30 mM glucose and cultured for 24 h. Then, pancreatic β-cell apoptosis was monitored using the TUNEL assay. As shown in Figure 1A, high glucose did not significantly modify the number of apoptotic nuclei, as assessed using chromatin morphology, with respect to the normal glucose (5 mM) group. In contrast, in the RIN-m5F cells exposed to taxol, used as a positive control, an intense red signal was observed in the cells with condensed and fragmented chromatin (white arrows) in the TUNEL. The increase in apoptotic β cells was only statistically significant with taxol, as shown in the graph (Figure 1A). To confirm these findings, we quantified the cell death with flow cytometry using an annexin V/7-AAD assay. Population diagrams did not show differences between the RIN-m5F cells incubated with 5 mM glucose and 30 mM glucose, but in cells treated with taxol, a significant increase in apoptosis was observed (Figure 1B). The early, late, and total apoptotic cells were quantified after 30 mM of glucose exposure and did not show any changes, as was seen for taxol, used as a positive control of apoptosis (Figure 1B). We next performed experiments to evaluate the cleaved-caspase-3, which is known as an executioner caspase in apoptosis. Western blot analysis indicated that the amount of caspase-3 did not show significant differences after high-glucose treatment; nevertheless, in the positive control with taxol, a significative increase in active caspase-3 was detected (Figure 1C). Therefore, these results indicate that high glucose, at this duration and concentration, did not induce apoptosis in the RIN-m5F cells. Finally, we also measured the cell viability with an MTT assay. It is well documented high glucose concentrations increase cell metabolism leading to an increase in NADH and NADPH levels, which together with dehydrogenases from metabolically active cells reduce tetrazolium to formazan in MTT assay [36]. Therefore, the results show that cell metabolism was increased by 40% after 24 h in β cells treated with high glucose compared to that in the control cells. In contrast, cell viability in the taxol group was significantly decreased (Figure 1D). Together, these results indicate that high glucose did not promote RIN-m5F cell apoptosis.

### 3.2. Silencing of Cx30.2 Increases Cell Apoptosis in RIN-m5F Cells

**Figure 2:**
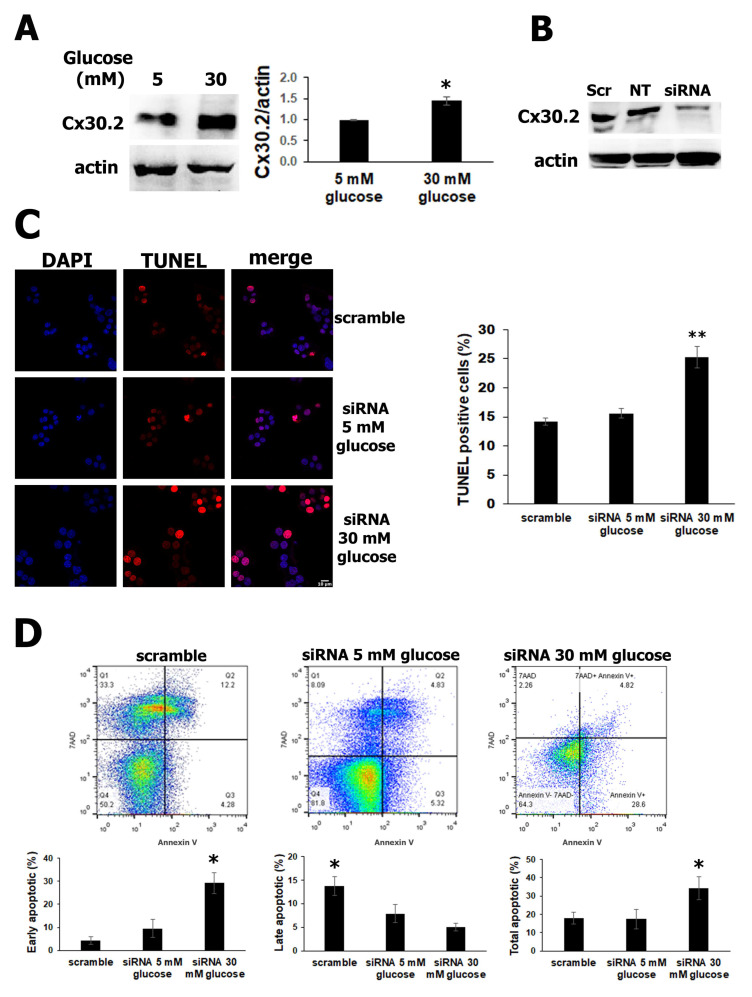
Increased Cx30.2 expression has a protective role in cell survival of RIN-m5F cells. (**A**) Western blot images show significantly augmented Cx30.2 (1.45-fold) with high glucose (30 mM) versus normal glucose (5 mM) and decreased Cx30.2 siRNA (**B**). (**C**) TUNEL assays detected a significant increase in positive apoptotic cells (red) in RIN-m5F cells with Cx30.2 siRNA with high glucose compared to Cx30.2 siRNA in normal glucose or scramble siRNA. (**D**) Flow cytometry using an annexin V kit detected an increase in early apoptosis in Cx30.2 siRNA cells incubated with high glucose versus normal glucose or scrambled siRNA. The data show the mean values ± SE. * *p* ˂ 0.05 and ** *p* ˂ 0.01 represent significant differences among the treatments from three independent experiments.

Cxs are essential proteins that play a critical role in the cellular intercommunication of β cells, promoting their homeostasis. Thus, the deletion of specific Cx36 results in a loss of the β-cell mass in the pancreatic islets, indicating that Cx36 could protect β cells against apoptosis [37]. To further explore the effect of high glucose on Cx30.2 expression, we grew β cells in high glucose (30 mM) for 24 h. The levels of Cx30.2 were analyzed using Western blotting. High glucose significantly increased the expression of Cx30.2 protein in β cells, by 1.45-fold (Figure 2A). In this work, we asked if Cx30.2 could be involved with β-cell survival. To gain insights, we examined whether the reduction of Cx30.2 expression has a role in apoptosis protection. Using siRNAs targeted against Cx30.2, we observed more than 65% efficiency of Cx30.2 knockdown in the β cells. In contrast, a significative reduction of Cx30.2 was not observed in the scramble siRNA control (Figure 2B). By silencing Cx30.2 in RIN-m5F cells with siRNAs and incubating them with high glucose, we found an increase in the red positive signal of TUNEL compared to cells transfected with the scrambled siRNA control or Cx30.2 siRNA in normal glucose (Figure 2C). Similar data were found for apoptosis measured using flow cytometry (Figure 2D), since the effect of silencing Cx30.2 produces a significant increase in the early and total apoptosis in Cx30.2 siRNA with high glucose versus Cx30.2 siRNA with normal glucose cells or scramble siRNA (*p* < 0.05).

### 3.3. Increased Cx30.2 Protein Expression from High Glucose Levels Does Not Change GJIC Activity

**Figure 3:**
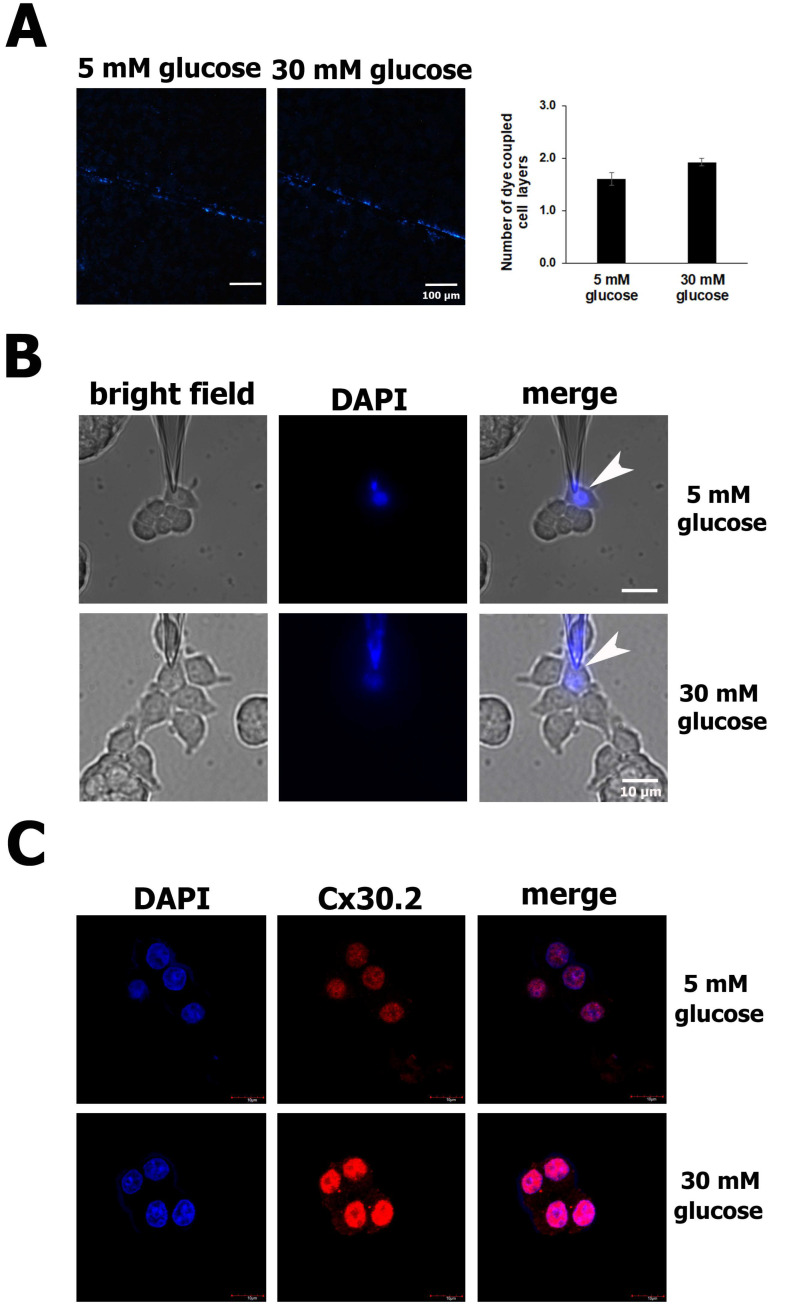
Increased Cx30.2 protein in high glucose did not change the GJIC activity in RIN-m5F cells. (**A**) Representative images from the SL-DT assay show that intercellular communication was not altered in cells exposed to high glucose (30 mM) compared to normal glucose (5 mM). In both conditions, DAPI was transferred at the same number of cell layers. (**B**) In the dye transfer assays, RIN-m5F cells were patch-clamped and dialyzed with DAPI dissolved in physiological internal solution in low- and high-glucose conditions. The images show no transfer of DAPI to any neighboring cell after 10–20 min in either the low- or high-glucose condition. (**C**) Confocal immunofluorescence images of a 1 µm thickness detected Cx30.2 proteins predominantly in nuclei stained with DAPI. All assays were carried out at least three independent times. The data show the mean values ± SE among the treatments from three independent experiments.

Finally, we wondered if the protection conferred by Cx30.2 against the apoptosis of RIN-m5F cells exposed to high glucose for 24 h was due to an increase in the intercellular communication between these cells. A scrape-loading-dye transfer (SL-DT) assay was used to assess the GJIC activity. Previously, it has been reported that GJ channels formed by Cx30.2 are permeable to cationic fluorescent dyes, such as ethidium bromide and DAPI [38,39]. Therefore, in this study, DAPI was used to analyze the permeability of RIN-m5F cells exposed to normal and high glucose concentrations for 24 h. The results from the SL-DT assays show no clear differences in the number of RIN-m5F cell layers diffused by the DAPI in normal glucose compared to the high glucose (Figure 3A). To confirm this, we performed dye transfer assays; however, after patch-clamping a cell and dialyzing it with the dye (white arrowheads), no transfer of DAPI to any neighboring cell was observed after 10–20 min (Figure 3B). Since high glucose did not affect the dye transfer between these β cells, we wondered if Cx30.2 was present in the cell membrane. To answer this question, we performed immunofluorescence assays to analyze the intracellular localization of Cx30.2 in RIN-m5F cells. Surprisingly, we found an intense positive signal for Cx30.2 in the nucleus in RIN-m5F cells incubated with 5 mM or 30 mM glucose, which explains why we did not detect DAPI dye transfer among these cells (Figure 3C). 

## 4. Discussion

Glucotoxicity is a significant condition that leads to pancreatic β-cell failure and eventually contributes to the development of several metabolic diseases, such as diabetes. It is well documented that chronic hyperglycemia has detrimental effects on pancreatic β-cell survival [40,41]. Specifically, it has been reported that high glucose exposure for 36 and 72 h induces apoptosis in RIN-m5F and INS-1 β cells, respectively [5,34,42]. However, the results of this study show that RIN-m5F β cells exposed to high glucose did not exhibit significantly increased cell apoptosis after 24 h. GJICs play key roles in maintaining cellular homeostasis and cell survival, where, through their structural components, Cxs may regulate insulin secretion in β cells. In this study, we showed that high glucose increased the Cx30.2 expression in β cells, conceivably as a mechanism to protect them against glucotoxicity exposure, since a reduction of Cx30.2 expression with Cx30.2 siRNA in high glucose markedly increased β-cell apoptosis compared to Cx30.2 siRNA in normal glucose in RIN-m5F cells. Therefore, this is the first evidence of Cx30.2 as an important regulator of β-cell survival under a glucotoxic environment.

On the other hand, it is well known that glucose metabolism may regulate the expression of many genes in β cells, such as Cx36, which was significantly reduced in insulin-secreting cell lines and primary isolated pancreatic rat islets by extracellular high glucose through the cAMP-PKA pathway [37]. Cx30.2 in RIN-m5F cells may also be modulated by this. Pieces of evidence indicate that the effects of high glucose on Cxs expression can be cell- or tissue-specific. For example, different results have shown that high glucose can repress both the mRNA and protein expression of Cx36, Cx30.2, and Cx43 in β cells, mouse pancreatic islets, and rat retinal endothelial cells, respectively [27,28,37,43]. However, opposite effects have also been found in different models, such as in the hearts of diabetic rats, H9c2 cardiac cells, and human retinal endothelial cells, where high glucose significantly increased Cx43 protein expression [44,45,46]. These results agree with the findings of the current study, indicating that high glucose increases the expression of Cx30.2. An increase in Cx30.2 expression may act as a mechanism against the stress condition imposed by high-glucose exposure. Several lines of evidence suggest that GJICs have a fundamental role in the control of cell death and survival. There are numerous examples of apoptosis stimulation through reduced GJIC activity. For instance, in pancreatic islets from mice exposed to toxic injuries, intercellular communication resulting from Cx36 was downregulated; in rat retinal Müller cells in high glucose, GJ communication mediated by Cx43 was decreased; in rat retinal endothelial cells silenced with Cx30.2 siRNA and in rat liver cells exposed to cadmium, GJIC activity was decreased [28,29,47,48]. These works demonstrate that cell-to-cell communication is necessary for cell survival. In contrast, GJIC inhibition using pharmacological agents was found to prevent apoptosis in other models, such as WB-MYC rat liver cells, NRK-52E cells, brain ischemia, and retinal neurons [49,50,51,52]. In this study, despite an increase in Cx30.2 expression, stimulated by high glucose in RIN-m5F cells, assays for GJIC activity suggest that the GJ channels formed by Cx30.2 were closed or not present in the cell membrane because they did not allow for the transfer of DAPI to neighboring cells, as previously documented [38,39]. Regarding the latter, in this work, the immunofluorescence assays showed that the Cx30.2 protein was surprisingly predominantly in the nuclei, which is consistent with the absence of dye transfer among the β cells. The localization of Cx30.2 in the nuclei suggests a non-canonical role of this Cx. In HeLa cells transfected with fragment C-terminal-Cx43, it was located in the nuclei, detecting decreased proliferation and suggesting that Cx43 may regulate gene expression [53]. Recent evidence indicated that the Cx43 carboxy tail was translocated to the nuclei of neural crest cells during their migration. In the nucleus, fragment of Cx43 form a complex with transcription factor 3 and polymerase II to bind to the N-cadherin promotor region to increase their transcriptional activity [54]. The above demonstrates that Cxs themselves can modulate the transcription of genes related to cell proliferation and cell death. 

Regarding the protection of Cxs beyond the GJ channel’s function, in glioma cells transfected with Cx43 and exposed to H_2_O_2_, this Cx was able to interfere with the pathway that triggers caspase-3 activation, inhibiting their apoptosis [55]. 

According to the growing evidence regarding the role of Cxs beyond their role in forming GJ channels, it is possible that Cx30.2 itself interacts in the nuclei with some transcription factor or signaling molecule, thus modulating the survival of β cells under glucotoxicity conditions. Further studies are needed to determine whether Cx30.2 in the nucleus leads to β-cell survival. 

## 5. Conclusions

This work establishes Cx30.2 as a new gene that is upregulated by high glucose after 24 h in β cells. Moreover, Cx30.2 has a protective role against apoptosis in glucotoxic stress environments in RIN-m5F β cells, and the protection mechanism conferred by Cx30.2 against apoptosis in high glucose is independent of its GJ channel function. 

## Data Availability

The original contributions presented in the study are included in the article, further inquiries can be directed to the corresponding authors.
